# A Systematic Review and Meta-Analysis of Randomized Trials to Evaluate the Impact of Exercise on Heart Rate Variability Post-Bariatric Surgery

**DOI:** 10.3390/jcdd11080248

**Published:** 2024-08-13

**Authors:** Tulio H. B. Bitencourt, Camila Marcondes de Oliveira, Andrey A. Porto, Davi C. de Andrade, David M. Garner, Rodrigo D. Raimundo, Vitor E. Valenti

**Affiliations:** 1 Autonomic Nervous System Center, Sao Paulo State University (UNESP), Marilia 17525-900, SP, Brazil; tulio.bitencourt@unesp.br (T.H.B.B.); cmarcondesom@gmail.com (C.M.d.O.); aa.porto@unesp.br (A.A.P.); davi.cavalheri@unesp.br (D.C.d.A.); 2 Cardiorespiratory Research Group, Department of Biological and Medical Sciences, Faculty of Health and Life Sciences, Oxford Brookes University, Headington Campus, Gipsy Lane, Oxford OX3 0BP, UK; davidmgarner1@gmail.com; 3 Laboratório de Delineamento de Estudos e Escrita Científica, Centro Universitário Faculdade de Medicina do ABC, Santo Andre 09060-870, SP, Brazil; rodrigo.raimundo@fmabc.br

**Keywords:** bariatric surgery, cardiovascular system, exercise, heart period, heart rate variability

## Abstract

Bariatric surgery is an approach used to treat patients with obesity in a small minority of eligible patients. Non-pharmacological therapies are important to maintain decent health status post-bariatric surgery. We performed a systematic review with meta-analysis to evaluate the effects of exercise on heart rate variability (HRV) in patients submitted to bariatric surgery. The searches were made via MEDLINE/PubMed (via the National Library of Medicine), EMBASE, Web of Science, and Scopus databases. We included non-blind, single-, or double-blind randomized control trials in patients older than 18 years of age submitted to bariatric surgery. The intervention group should be submitted to an exercise training protocol, including aerobic, strength, and other exercise modality after bariatric surgery. We documented 245 studies, and after screening and eligibility phases, only 4 were included. We observed no significant change for the SDNN: subtotal = 19.74 (CI: −4.98, 44.45), *p* = 0.12, I^2^ = 85% (very low quality of evidence); pNN50: subtotal = 13.09 (CI: −9.17, 35.35), *p* = 0.25, I^2^ = 93% (very low quality of evidence); RMSSD: subtotal = 8.44 (CI: −3.61, 25.50), *p* = 0.17, I^2^ = 95% (very low quality of evidence); SD1: subtotal = 9.36 (CI: −4.48, 23.21), *p* = 0.19, I^2^ = 96% (very low quality of evidence). We could not detect significant effects of exercise on resting HRV after bariatric surgery. The low certainty of the results via the evidence level analysis suggest further studies might be beneficial.

## 1. Introduction

Obesity can be defined as an excess of adipose tissue inducing bodily harm. It is a pandemic that continues to increase during global modernization [1]. Obesity is normally evaluated using the body mass index (BMI) [2]. There is a close connection between mortality or morbidity and increased BMI, indicating that higher BMI is clearly related to a higher probability of disease and subsequent mortality [3]. Previous data have stated that in the United States of America, the prevalence of obesity (BMI > 30 kg/m^2^) was almost 40% between 2015 and 2016 [4]. When considering severe obesity (BMI ≥ 40 kg/m^2^), it has already attained 7.7% amongst ethnic groups [5].

It was assessed that for every five-unit increase in BMI (above 25 kg/m^2^), the probability of all-cause mortality increased by around 29%, vascular mortality increased by almost 41%, and diabetes-related mortality reached 210% [6].

Considering the health risks of obesity, bariatric surgery seemed to be an approach to treat obesity in a small group of eligible patients [7]. Hitherto, the scientific research literature specified bariatric surgery as the most effective intervention for improvement of comorbidities and weight loss [8,9]. Nevertheless, adverse effects of bariatric surgery include nutritional deficiency [10] and increased fracture risk owing to reduced bone mineral density [11].

In this sense, clinical strategies to maintain a respectable health status after bariatric surgery are needed to improve quality of life and increase life expectancy [12]. Exercise is a non-pharmacological and inexpensive intervention that advances health status in numerous situations, including breast cancer patients [13], adolescents with mild and moderate idiopathic scoliosis [14], and psychological depression [15].

With this in mind, and with the purpose of monitoring the subjects’ health status, heart rate (HR) variability (HRV), which is the measurement of heartbeat oscillation and estimates the autonomic nervous system function [16], has been demonstrated to offer relevant clinical information about health status [17,18,19,20]. Thus, its use for evaluation of the quality of life helps us to better understand the patients’ health status.

Based on the aforesaid results, the relationship between HRV, bariatric surgery, and exercise is a matter of consideration in cardiovascular clinical research. As a consequence, we raised the following queries so as to better comprehend this relationship: Is there clinical evidence about the effects of exercise on HRV in post-bariatric surgery patients? Can we recommend exercise intervention for bariatric surgery patients to improve HRV? Furthermore, very recently, a systematic review concluded that exercise programs in post-bariatric surgery patients are not well reported [21]. Hence, this study estimated the effect of exercise on HRV in post-bariatric surgery patients, conducted using a systematic review and meta-analysis.

## 2. Materials and Methods

### 2.1. Registration

This systematic review followed the recommendations of the Preferred Reporting Items for Systematic Reviews and Meta-Analyses (PRISMA) [22] and was registered in the PROSPERO platform (CRD42023473219).

### 2.2. Search Strategy and Study Selection

The searches were performed via MEDLINE/PubMed (via the National Library of Medicine), EMBASE, Web of Science, and Scopus databases with the submission of the following keywords: ((“Stomach Stapling” OR “Bariatric Surgical Procedure” OR “Bariatric Surgery”) AND (“Exercise” OR “Physical Activity” OR “Physical Exercise” OR “Exercise Training” OR “Isometric Exercise” OR “Aerobic Exercise”) AND (“Autonomic Nervous System” OR “Vegetative Nervous System” OR “Visceral Nervous System” OR “Heart Rate Variability” OR “Heart Period”)).

We searched for the terms in the whole text, and all included keywords were provided in the MeSH Cochrane Library online system [23].

We exported all references to the Rayyan QCRI program (Qatar Computing Research Institute, Qatar) so as to exclude duplicates. The publications were screened in the Rayyan online software 2024 by analyzing the title and abstract. This stage was performed by two independent reviewers (THBB and AAP), who read the entire articles. One more reviewer (VEV) was invited to decide if there was a disaccord regarding a particular study.

### 2.3. Eligibility Criteria

The studies were published from the start of the databases until September 2023 and from peer-reviewed journals. The inclusion and exclusion criteria were compatible with the PICOS (Population, Intervention, Comparison, Outcomes, and Study Design) elements, including:

(P) Male or female post-bariatric surgery patients older than 18 years with or without associated diseases.
(I) The intervention group should be submitted to an exercise training protocol, including aerobic and/or strength exercise modality after bariatric surgery, in which the participants were submitted to a minimum of three sessions per week and lasted at least four weeks. We excluded publications that did not include aerobic or strength exercise.
(C) For comparison groups, we included studies that evaluated subjects that were not submitted to exercise or references that compared the patients vs. themselves.
(O) The outcome of interest is the HRV indices.
(S) We included studies with no blind, single-, or double-blind randomized control trials (RCTs). This investigation was limited to articles published in peer-reviewed journals and written in English. We excluded studies performed with animals, conference papers, master’s theses, doctoral dissertations, descriptive studies, case studies, editorials, and reviews.

### 2.4. Data Extraction

Data relating to the author, study design, study participants’ features, intervention, and exercise protocols were extracted from original studies and presented in Table 1. Missing data were obtained by contacting the study authors. This phase was completed by two independent reviewers (THBB and AAP). When the author’s correspondent did not reply, the Web Plot Digitizer^®^ was used to calculate the data presented in figures. The HRV data were charted as mean and standard deviations (SD). Values presented with “standard error” or “confidence intervals” (CI) in the primary studies were then changed to SD.

### 2.5. Assessment of the Risk of Bias

The analysis of bias was shaped by Risk of Bias tools originated in the Cochrane organization [28] via the Review Manager program (RevMan 5.4.1). Risk of bias is a tool based on the domains. Its evaluation was divided into six areas: “randomization process”, “deviations from intended interventions”, “missing outcome data”, “measuring of the outcome”, “selection of the reported results”, and “overall bias”. The cataloging was divided into three retorts: low risk, some concerns, and high risk. Our assumptions were based on the table developed in [28], “Reviewers’ judgment and criteria for judgment”. Two independent authors finished the risk of bias analysis (THBB and AAP). An additional researcher (VEV) was consulted in case of discrepancies in their judgements. The assessors of the risk of bias were trained with appropriate sessions (THBB, VEV, AAP, and RDR).

### 2.6. GRADE (Levels of Evidence)

We enforced the Grades of Recommendation, Assessment, Development, and Evaluation (GRADE) Working Group (GRADE Working Group, 2004) to measure the certainty of the evidence. This analysis comprised the study design of randomized trials (strong evidence). We similarly considered study quality (detailed study methods and execution) and limitations in the strength of evidence analysis [29]. We completed the GRADEpro GDT v4^®^ (McMaster University, Hamilton, ON, Canada) to elaborate the summary of the findings table.

### 2.7. Qualitative Analysis (Systematic Review)

A narrative synthesis was executed to describe detailed data on how each study was completed. The details for each study were introduced in texts and tables. The results of the individual qualitative analysis per study were accomplished by studying HRV parameters for the intervention or control protocols.

### 2.8. Quantitative Analysis (Meta-Analysis)

After selecting all references, we estimated the possibility of a meta-analysis. In a positive situation, we introduced the HRV values. The information required to construct the meta-analysis was the pre- and post-intervention period. We assumed the criterion of extracting all data offered between protocols in pre- and post-intervention.

Heterogeneity was considered using the I^2^ statistic. We interpreted high heterogeneity if I^2^ < 50%, moderate for I^2^ between 50% and 75%, and low for I^2^ > 75% [30,31]. For the “95% CI” and “test for overall effect size” values, significant differences were assumed for *p* < 0.05 (or <5%). We computed the absent SDs of the changes (SDchanges) if the publications did not present dispersion values of change, such as 95% confidence intervals, SD, *p*-values, or standard errors. We stated the results of the meta-analysis in weighted mean difference (MD), 95% CI, and *p*-value. We considered *p* < 0.05 (or <5%) statistically significant for the overall MD of the intervention group compared to the control group. The results were presented as forest plots. We executed a random-effect model, as this is a more conservative technique that permits the study heterogeneity to deviate beyond chance, providing further generalizable results [32]. All data were formed using the Review Manager Program (RevMan 5.4.1).

## 3. Results

### 3.1. Description of Studies

We recognized a total of 245 publications. After eliminating duplicates (n = 155), 90 references were screened for inclusion. Among the studies, 83 records were excluded after reviewing the title and/or abstract and 1 study was not retrieved. Three studies were eliminated because they were abstracts presented in Congress.

**Figure 1:**
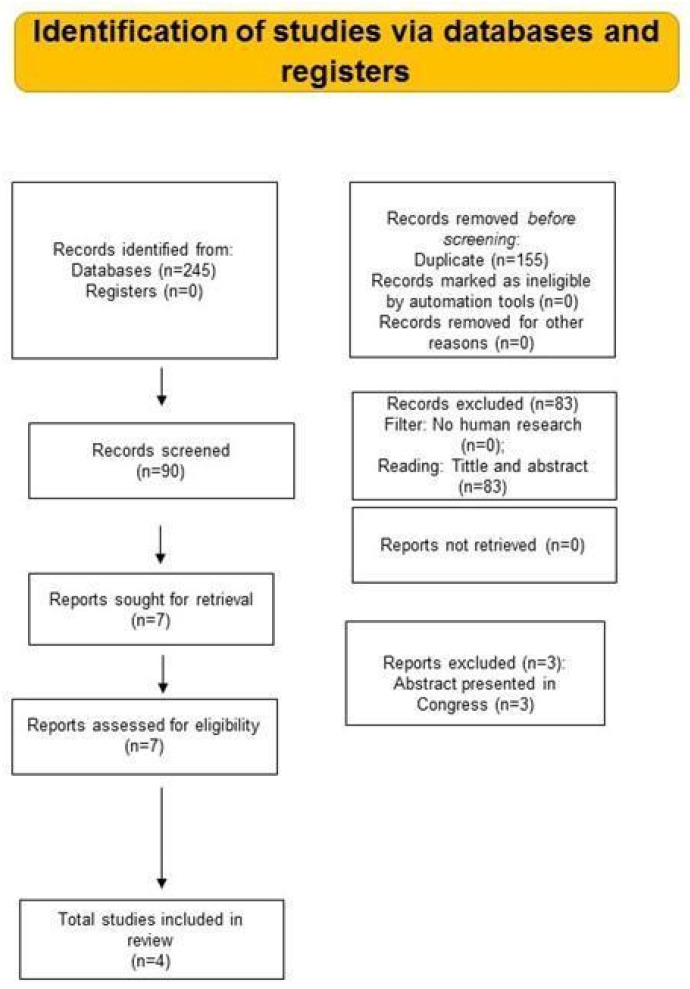
PRISMA 2020 flow diagram.

Four papers were designated for full-text reading, GRADE, risk of bias assessment, and qualitative analysis [24,25,26,27]. The study features included are illustrated in Table 1. All references [24,25,26,27] were included for meta-analysis. The search method and selection stages were in accordance with the PRISMA protocol flow diagram (Figure 1). 

The studies contained within this review were published between 2011 and 2022 (Table 1). Three studies originated from Brazil [29,30,31] and one from Canada [27]. Three studies were presented as randomized and controlled trials [29,30,31,32] and one study as a randomized, double-blinded, sham-controlled clinical trial [27].

Castello et al. (2011) included patients with obesity (men) with body mass index (BMI) ≥ 40 kg/m^2^, and Castello-Simões et al. (2013) also investigated patients with obesity (women; BMI ≥ 40 kg/m^2^). Ricci et al. (2020) inspected patients with obesity (men and women; BMI > 30 kg/m^2^) and Belzile et al. (2022) investigated just patients with severe obesity (BMI ≥ 40 kg/m^2^). Mean age was similar between the references: 20 to 45 years—training group: 38 ± 4 years vs. control: 36 ± 4 years (Castello et al., 2011); 20 to 45 years—training group: 32 ± 4 years vs. control: 31 ± 2 years (Castello-Simões et al., 2013); 18 to 50 years—training group: 40.7 years (36.2–45.4) vs. control: 37.6 years (32.4–42.8; Ricci et al., 2020); training group: 41.6 ± 11.6 years vs. control: 42.3 ± 10.8 years (Belzile et al., 2022) [24,25,26,27,32].

Regarding exclusion criteria, Castello et al. (2011) [24] excluded patients with neurological or orthopedic diseases that circumvented them performing an exercise program, implanted pacemaker, chronic dysfunction in heart rhythm, myocardial infarction, unstable angina, significant acute arrhythmias, valvular heart disease, uncontrolled hypertension, previous history of heart disease, uncontrolled diabetes mellitus, chronic obstructive pulmonary disease, associated surgery, post-menopause, beta-blocker treatment, and current regular exercise program involvement.

Castello-Simões et al. (2013) [25] excluded patients with neurological or orthopedic disorders, cardiac arrhythmias, ECG alterations, previous history of heart disease, uncontrolled hypertension, uncontrolled diabetes mellitus, chronic obstructive pulmonary disease, β-blocking pharmacotherapy, post-menopause, and those currently partaking in a regular exercise program.

Ricci et al. (2020) [26] excluded subjects with neurological or orthopedic disorders that stopped them from performing the physical exercise program, implanted pacemaker or any metal prosthesis, Baecke physical activity questionnaire [33] score > 8, insulin-dependent diabetes mellitus, hypertension, heart disease history, beta-blocker treatment, contraindications to cardiopulmonary exercise testing, respiratory diseases, distal arteriopathy, hypersensitivity to electrostimulation, inflammatory, hepatic, and renal dysfunctions, diabetic neuropathy, cognitive deficit, deficiency in adhering to the study protocols, illicit drug use, and pregnant or post-menopausal women.

Belzile et al. (2022) [27] excluded patients who had earlier bariatric surgery, subjects acquiesced to vagotomy procedure, subjects who were taking weight-loss medications three months prior to the experiments, and patients with an implanted pacemaker.

Concerning interventions, the sessions conducted by Castello et al. (2011) [24] persisted for one hour on different days: 3 times a week, for 12 weeks (36 sessions in total). Each session comprised of 5 initial minutes of upper and lower limb stretching in front of a mirror in a standing and sitting position, and a 5 min warmup on a treadmill at 3 km/h for 40 min, with pace and slope varying according to HR. The 40 min of exercise were separated into 4 stages of 10 min each:

1. Exercise intensity (50% of the HR peak),
2. 60% of peak HR,
3. 70% of peak HR,
4. Maintenance of 70% peak HR.

Next, the subjects were submitted to 1 min of recovery at 3 km/h and 10 min of the same initial stretch and diaphragmatic breathing.

The sessions conducted by Castello-Simões et al. (2013) [25] were performed for one hour on different days: three times/week, for twelve weeks. The intervention was based on an initial 5 min of upper and lower limb stretching, and a 5 minutes warmup on a treadmill at 3 km/h for 40 min, with pace and inclination extending according to HR. The 40 min of exercise were separated into 4 stages (10 min each):

1. Exercise intensity (50% of HR peak),
2. 60% of HR peak,
3. 70% of HR peak,
4. Maintenance of 70% HR peak.

After that, the patients were submitted to 1 min of recovery at 3 km/h and 10 min of the same initial stretching.

The training protocol led by Ricci et al. (2020) [26] consisted of 30 sessions, 5 times/week, completed over 6 weeks. The whole-body electromyostimulation protocol was achieved with an electromyostimulator. The electrical current intensity was nominated for each location and gradually modified throughout each session to improve the intensity of stimulation. The exercises were achieved concurrently with the electric current on—it was completed with no loads in the orthostatic position. The exercises combined the following movements: trunk flexion, squat, isometric abdomen contraction, and exercises of upper limbs.

Exercise training conducted by Belzile et al. (2022) [27] was initiated three months after bariatric surgery and was performed three days/week for twelve weeks. The training was separated into:

1. 35 min of moderate to strong aerobic exercise (60–75%—HR reserve),
2. 25 min of resistance exercises. Loads were not provided.

### 3.2. Qualitative Analysis

Castello et al. (2011) [24] stated that aerobic exercise training improved HRV in women who underwent bariatric surgery. SDNN, RMSSD, NN50, pNN50, SD1, and SD2 HRV indices were significantly increased after a 12-week aerobic exercise program in the training group, whilst no significant change was demonstrated in the control group. Furthermore, the post-training HRV indices (SDNN, RMSSD, NN50, pNN50, SD1, and SD2) were greater in the training group compared to the control group. HRV was analyzed four weeks after bariatric surgery.

According to Castello-Simões et al. (2013) [25], HRV was improved after a 12-week aerobic training protocol in women who underwent bariatric surgery. Then, the authors detected that both RMSSD and SD1 indices increased after the exercise program in the training group, whereas no significant change was reported in the control group. Post-exercise SD1 was significantly greater in the training group compared to the control group. HRV was similarly studied four weeks after bariatric surgery. Besides, Belzile et al. (2022) [27] described that all time domain indices (SDNN, SDANN, pNN50, and RMSSD) significantly improved in the exercise group.

Equally, Ricci et al. (2020) [26] failed to locate a significant outcome of whole-body electromyostimulation exercise on linear HRV in bariatric surgery patients. RMSSD, RRTri, LF (n.u.), HF (n.u.), SD1, and SD2 were not changed amid pre- and post-training exercises in the training group. Yet, RMSSD, RRTri, SD1, and SD2 HRV indices were significantly increased in the control group. When studying nonlinear HRV, the authors recognized that approximate entropy and sample entropy increased, while Shannon entropy decreased following exercise training in the training group, despite the fact that no significant changes were detected in the control group. The patients were evaluated seven weeks following bariatric surgery.

### 3.3. Analysis of the Risk of Bias

**Figure 2:**
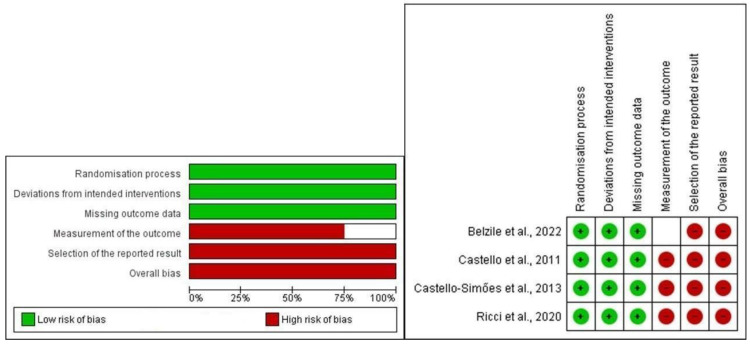
Cochrane risk of bias tool [24,25,26,27].

We found different results during the risk of bias analysis among the four references. The evaluation of the risk of biased is summarized in Figure 2. Additional data regarding the risk of bias are provided in the Supplementary Materials: risk of bias findings about each risk of bias item for each included study.

#### 3.3.1. Randomization Process

All studies (100%) clarified events for conducting the allocation randomization sequence. The allocation sequence was hidden until participants were enrolled and assigned to their interventions.

#### 3.3.2. Deviations from Intended Interventions

In the four studies, all participants (100%) were conscious of their intervention throughout the trial, as it was impossible to blind the exercise intervention. There was no evidence that the intended intervention arose due to the trial environment, and an adequate analysis was used to calculate the intervention effect.

#### 3.3.3. Missing Outcome Data

All references (100%) [24,25,26,27] clarified that outcome data were unconnected to the trial context. Data for this outcome were available for all, or nearly all (at least 95%), participants randomized in no study (0%). In three references, sample loss was based on patients that declined to continue—it was not linked to their health status.

#### 3.3.4. Measurement of the Outcome

HRV measurement was suitable in three studies (100%). Measurement of the consequences did not vary between intervention groups in the three references (100%). Outcome assessors were conscious of the intervention received by study participants in the three references (100%).

#### 3.3.5. Selection of the Reported Result

Throughout all three studies (100%), the data that formed the result were in keeping with a pre-specified analysis plan that was confirmed before unblinded outcome data were available for analysis.

#### 3.3.6. Overall Bias

Considering all items, the result was high risk of bias for the three references (100%).

### 3.4. Quantitative Analysis

**Figure 3:**
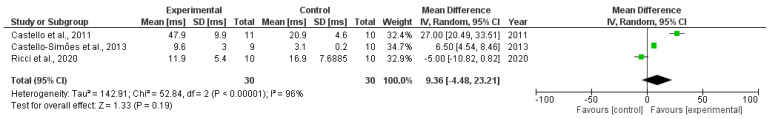
Meta-analysis for overall effects of exercise on the SD1 HRV index in bariatric surgery patients in the “exercise” subgroup. HRV: heart rate variability; SD1: short-term variation in the RR intervals [24,25,26].

**Figure 4:**
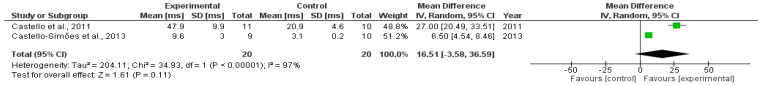
Meta-analysis for overall effects of exercise on the SD1 HRV index in bariatric surgery patients in the “aerobic exercise training with no additional stimulation” subgroup. HRV: heart rate variability; SD1: short-term variation in the RR intervals [24,25].

**Figure 5:**
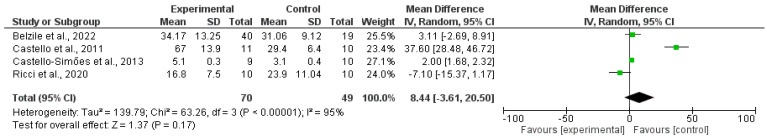
Meta-analysis for overall effects of exercise on the RMSSD HRV index in bariatric surgery patients in the “exercise” subgroup. HRV: heart rate variability; RMSSD: root mean square of the successive differences [24,25,26,27].

For the SD1 and RMSSD results, we performed a random effect and mean difference (MD) model to measure effect sizes. The black diamond indicates the 95% CI (Figure 3, Figure 4 and Figure 5). A negative effect size indicates upgraded values in the intervention group compared to the control (no exercise).

#### 3.4.1. SD1

With regard to SD1, no significant change was distinguished for this HRV index. In the “test for overall effect”, we revealed a subtotal = 9.36 ms (95%CI: −4.48, 23.21), *p* = 0.19, and heterogeneity = 96% (Figure 3). In the subgroup “aerobic exercise training with no additional stimulation”, there were no significant changes in the “test for overall effect”: subtotal = 16.51 ms (95% CI: −3.58; 36.59), *p* = 0.11, and heterogeneity = 97% (Figure 4). The GRADE quality of evidence for this result was very low (Table 2).

#### 3.4.2. RMSSD

**Figure 6:**
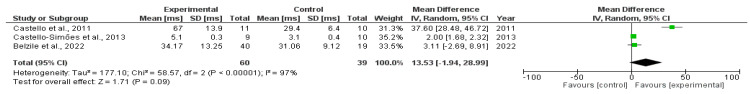
Meta-analysis for overall effects of only aerobic exercise on the RMSSD HRV index in bariatric surgery patients in the “aerobic exercise training with no additional stimulation” subgroup. HRV: heart rate variability; RMSSD: root mean square of the successive differences [24,25,27].

We noted that no significant change was recognized for the RMSSD. In the “test for overall effect”, we revealed a subtotal = 8.44 ms (95%CI: −3.61, 20.50), *p* = 0.17, and heterogeneity = 95% (Figure 5). In the subgroup “aerobic exercise training with no additional stimulation”, there were no significant deviations in the “test for overall effect”: subtotal = 13.53 ms (95% CI: −1.94; 28.99), *p* = 0.09, and heterogeneity = 97% (Figure 6). The GRADE quality of evidence for this result was very low (Table 2).

#### 3.4.3. pNN50

**Figure 7:**
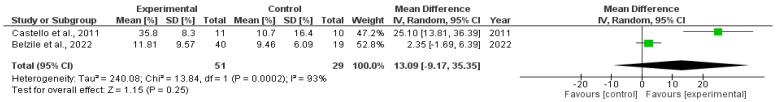
Meta-analysis for overall effects of exercise on the pNN50 HRV index in bariatric surgery patients in the “exercise” subgroup. HRV: heart rate variability; pNN50: percentage of adjacent RR intervals with a difference in duration greater than 50 ms [24,27].

Regarding the pNN50 index, we observed no significant change. In the “test for overall effect”, we revealed a subtotal = 13.09 ms (95%CI: −9.17, 35.35), *p* = 0.25, and heterogeneity = 93% (Figure 7). The GRADE quality of evidence for this result was very low (Table 2).

#### 3.4.4. SDNN

**Figure 8:**
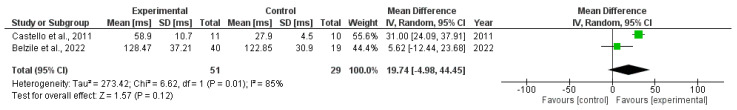
Meta-analysis for overall effects of exercise on the SDNN HRV index in bariatric surgery patients in the “exercise” subgroup. HRV: heart rate variability; SDNN: standard deviation of all normal RR intervals recorded in a time interval [24,27].

In regard to the SDNN, we revealed no significant change. In the “test for overall effect”, we revealed a subtotal = 19.74 ms (95%CI: −4.98, 44.45), *p* = 0.17, and heterogeneity = 85% (Figure 8). The GRADE quality of evidence for this result was very low (Table 2).

## 4. Heterogeneity

The very high heterogeneity reported for SD1, RMSSD, pNN50, and SDNN HRV indices (I^2^ between 85% and 97%) may be explained by the clinical and methodological diversity between studies. Although the sample profile was similar between the references (obese men and women 30–50 years old), we detected differences in the exercise protocols since in one study, the patients were submitted to strength and aerobic exercise [27]. In addition, one publication [24] lacked information related to the control group (Table 1). We also identified variability in risk of bias because measurement of the outcome was different in one study [27] (Figure 2).

## 5. Discussion

This systematic review was commenced to evaluate the effects of exercise on HRV in bariatric surgery patients. As key findings, we reported the following:

(1) According to the meta-analysis, no significant effect of exercise on HRV was recognized.
(2) The GRADE quality of evidence evaluation for SDNN, pNN50, SD1, and RMSSD presented very low certainty.
(3) Risk of bias assessment emphasized concerns regarding blind assessment and further prejudices or bias.

The effects of bariatric surgery on HRV were reviewed by our group [34]. The 14 selected references in the cited study permitted us to indicate that bariatric surgery improves HRV. Considering that proinflammatory cytokines produced by excess adipose tissue induce higher sympathetic activity, leading to HRV impairment [35,36,37,38], the theory is that the cytokine reduction owing to decreases in adipose tissue increases parasympathetic system activity, and HRV as a result.

In this situation, earlier studies have stated that exercise training provides advantageous effects on bariatric surgery [39,40,41]. Resistance training combined with protein supplementation in patients after bariatric surgery enhanced physical activity and cardiorespiratory fitness, reducing cardiovascular and metabolic risk. Oppert et al. (2018) [40] introduced patients to exercise six weeks after bariatric surgery. The exercise training program was founded on leg extension, abdominal crunch, leg press, chest press vertical traction, and bicep curls. Each exercise was conducted in 8 to 12 repetitions, 4 times. Exercise intensity began with eight repetitions at 50% of one repetition maximum. Repetitions enhanced from 8 to 9 to 12, and loads increased from 50% to 65% to 75% of 1 maximum repetition. Resting between repetition persisted for around 60 s.

The review published by Bellicha et al. (2021) [39] showed that exercise programs after bariatric surgery improved muscle strength and cardiorespiratory fitness. Their study similarly suggested that exercise interventions are able to lessen bone loss and prevent weight regain after surgery, which is relevant to increased quality of life [41,42]. Together, we were anticipating that a systematic review with meta-analysis could reveal significant benefit of exercise on HRV following bariatric surgery.

Even so, our quantitative analysis failed to detect a significant positive impact of exercise on post-bariatric surgery. The methodological procedures conducted by the selected references [24,25,26,27] permitted us to complete a meta-analysis only for SDNN, pNN50, RMSSD, and SD1 HRV indices, which provided information related to the estimation of parasympathetic influence on HR oscillations (pNN50, RMSSD, and SD1) and global HR control (SDNN) [17].

For the RMSSD and SD1 results, we separated measurements into “exercise”, including aerobic exercise and whole-body electromyostimulation associated with dynamic exercise, and “aerobic exercise training with no additional stimulation”, excluding whole-body electromyostimulation associated with dynamic exercise. With regard to the RMSSD index, in the subgroup “exercise”, the exercise intervention groups sustained a mean SD = 10.56 ms (95% CI: −9.44, 30.55), higher than the control group after the exercise intervention period (*p* = 0.3). The “aerobic exercise training with no additional stimulation” subgroup showed 19.5 ms (95% CI: −15.39, 54.38), higher than the control group following the exercise program period (*p* = 0.27).

Concerning the SD1 result, in the “exercise” subgroup, the exercise intervention groups sustained a mean SD = 9.36 ms (95% CI: −4.48–23.21), higher than the control group after the exercise intervention period (*p* = 0.19). In the “aerobic exercise training with no additional stimulation” subgroup, the exercise intervention groups sustained a mean SD = 16.51 ms (95% CI: −3.58, 36.59), higher than the control group after the program intervention period (*p* = 0.11).

The mentioned results indicate that parasympathetic control of HR was unaffected by exercise intervention in post-bariatric surgery patients. This is because SD1 and RMSSD are influenced by vagal nerve activity [40,41].

Regarding SDNN and pNN50, there was no need to investigate a subgroup, as all three references performed aerobic exercise as an intervention [24,25,27]. When evaluating the SDNN index, the exercise intervention groups sustained a mean SD = 19.74 ms (95% CI: −4.98, 44.45), higher than the control group after the exercise intervention period (*p* = 0.12). In the pNN50 analysis, we detected a mean of 13.09 ms (95% CI: −9.17, 35.35), higher than the control group following the exercise program period (*p* = 0.25).

In this case, pNN50 provides information regarding vagal control of HR, while SDNN is related to both sympathetic and parasympathetic regulation of HR [16,17].

As a consequence, based on the above-mentioned data, we suggest that there is no solid evidence that exercise improves resting HRV after bariatric surgery in patients with obesity.

Alternatively, we initially supposed that the exercise group would show significantly higher HRV values after the intervention period compared to the control groups. However, while two references [24,25] exhibited a significant positive effect of exercise on HRV after bariatric surgery, no significant difference was noted in the meta-analysis. This is possibly a result of the large differences in resting HRV between the three studies. We understand that the discrepancy between Castello et al. (2011) [24] and Castello-Simões et al. (2023) [25] regarding resting RMSSD and SD1 indices had a critical effect on the meta-analysis.

It is worth remarking that an earlier study raised solid evidence indicating homology between RMSSD and SD1 [43]. It was established via empirical and mathematical methods that RMSSD and SD1 deliver identical metrics. The main apprehension is that the inappropriate use of both indices may affect the interpretation of HRV since it could provide redundant data.

In this context, unexpected data reported by Ricci et al. (2020) [26] indicated that HRV was increased in the sham group. Consistent with their results, RMSSD, triangular index (RRTri), SD1, and SD2 indices were higher after a sham intervention compared to baseline, whereas no significant change was recognized between baseline HRV vs. HRV after intervention in the experimental group regarding all linear HRV indices. This may be because the sham group likewise performed dynamic exercise (trunk flexion, squat, isometric abdomen contraction, and exercises of upper limbs) [42], which may have influenced resting HRV.

The four selected references [24,25,26,27] did not signal any problems regarding events for generating the allocation randomization sequence. These studies’ methodological descriptions indicated that the allocation sequence was hidden until participants were enrolled and assigned to their interventions. Still, a pertinent fact that our review highlighted was that patients and outcome assessors were conscious of their assigned intervention throughout the trial, as it was impossible to blind the exercise intervention. In contrast, we recognized no indication that the intended intervention arose owing to the trial context, and an appropriate analysis was necessary to estimate the assignment to intervention effect. In addition, sample loss in the four references was attributable to patients that refused to continue—it was not related to their current health status.

We also drew attention to the measurement of the outcome and selection of the reported result as possible bias in the selected references. Considering that outcome assessors were aware of the intervention, this may be related to flaws in estimating the participants’ variables that arose when the values were not similar to the accurate data [28]. Additionally, we detected limitations in the selection of the reported result. This item indicates if the researchers’ pre-specified intentions were available in sufficient detail and may indicate unexpected changes during the trial [28].

Regarding the clinical implication of our study, we need to recognize that although our systematic review did not find a significant effect of exercise on HRV in post-bariatric surgery patients, it does not mean that exercise is not recommended to this population. HRV is not the only health marker that can be followed by the clinical team, many other variables can also be followed, such as weight, blood pressure, blood cholesterol, glucose and triglyceride levels, anthropometric parameters, adipose tissue, fat mass percentage, and inflammatory markers.

Moreover, the beneficial effects of exercise are well demonstrated for symptoms related to obesity [44], including depression [45], elevated visceral fat [46], increased waist circumference [47], insulin resistance [48], and cardiometabolic diseases [49]. Indeed, our data did not recommend for the clinical team to avoid exercise interventions in post-bariatric surgery patients.

Our analysis emphasized several concerns: Levels of evidence analysis achieved through GRADE indicated very low certainty for all HRV indices evaluated in the meta-analysis (RMSSD, SDNN, SD1, and pNN50). This was by reason of high heterogeneity, large 95% CI, and high risk of bias in at least one reference. The exercise intervention started one month after bariatric surgery in three references [24,25,26], while it started three months after bariatric surgery in one study [27], which may have prejudiced the intervention standardization in the meta-analysis interpretation. We selected a small number of references, and this is an important limitation in our study since it restricts interpretation and clinical practice. Nonetheless, we assessed only a few studies because we aimed to include only randomized control trials, which is the gold standard when analyzing clinical practice [50]. The meta-analysis included a small number of studies, which may affect statistical comprehension. Conversely, according to Cochrane recommendations, meta-analysis is valid for statistical combinations of results from two or more separate studies [30,31]. Three [24,25,26] out of the four selected references were published by the same group, and this may influence the interpretation for different populations worldwide. We extracted data from graphs in one study through the Web Plot Digitizer, which may have influenced the accuracy.

Taken together, we recommend future randomized clinical trials in post-bariatric surgery with aerobic and strength exercise modalities focused on HRV time and frequency domain parameters, which will support us to better understand the effects of exercise on this outcome. We also strongly suggest the authors follow the Consolidated Standards of Reporting Trials (CONSORT) statement [50] and consult systematic review instruments, such as the risk of bias and GRADE tools, to plan the randomized clinical trials.

Another issue to be highlighted is the exercise intensity, which extended between 60% and 75% in the aerobic exercise protocols [24,25,26,27], while loads for the resistance exercise training were not provided by Belzile et al. (2022) [27], and Ricci et al. (2020) [26] conducted dynamic exercises with no loads in the orthostatic position. Also, HRV analysis was resultant from a 24 h period in the work of Belzile et al. (2022) [27], but Castello et al. (2011) [24], Castello-Simões et al. (2013) [25], and Ricci et al. (2020) [26] evaluated HRV via a shorter time-series, under one hour.

## 6. Conclusions

In conclusion, our systematic review with meta-analysis did not find significant effects of exercise on resting HRV after bariatric surgery. Furthermore, no decision could be made owing to the low certainty of the results via the evidence level analysis. Consequently, we recommend further research studies.

## Figures and Tables

**Table 1:** Characteristics of the study populations of articles by author and year, sample, age (years), intervention details, control, and outcomes measured.

Author/ Years	Study Design	Sample Size	Age (Years)	Intervention Details	Control	Outcomes Measured
Castello et al., 2011 [24]	Randomized controlled trial	Obese women Training group: 11 Control group: 10	Training group: 38 ± 4 Control: 36 ± 4	Exercise onset: 1 month after bariatric surgery. Duration: 3 days/week for 12 weeks. Protocol: 5 min stretching, 5 min warmup on a treadmill (3 km/h), 10 min at 50% of HR peak, 10 min at 60% of HR peak, 10 min at 70% of peak HR, recovery for 1 min (3 km/h), and 10 min of stretching.	Control intervention was not explained.	All HRV indexes (SDNN, RMSSD, pNN50, NN50, SD1, and SD2) improved in the training group.
Castello-Simões et al., 2013 [25]	Randomized controlled trial	Obese women Training group: 9 Control group: 10	Training group: 32 ± 4 Control: 31 ± 2	Exercise onset: 1 month after bariatric surgery. Duration: 3 days/week for 12 weeks. Protocol: 5 min of stretching, 5 min warmup on a treadmill (3 km/h), 10 min at 50% of HR peak, 10 min at 60% of HR peak, 10 min at 70% of peak HR, recovery for 1 min (at 3 km/h), and 10 min of stretching.	The control group did not practice any physical activity.	RMSSD and SD1 HRV indices improved in the training group.
Ricci et al., 2020 [26]	Randomized, double-blinded, sham-controlled clinical trial	Obese women and men Training group: 10 Control group: 10	Training group: 40.7 (36.2–45.4) Control: 37.6 (32.4–42.8)	Exercise onset: 8 days after bariatric surgery. Duration: 5 days/week for 6 weeks. Protocol: 10 different types of movements: squat, trunk flexion, exercises of upper limbs, and isometric abdomen contraction with an electromyostimulation apparatus and no load.	The sham group performed the same exercises with no electric current.	Not all HRV indices (RMSSD, RRTri, LF, HF, SD1, SD2, ApEn, SampEn, and Shannon) were changed in the training group.
Belzile et al., 2022 [27]	Randomized controlled trial	Obese women Training group: 40 Control group: 19	Training group: 41.6 ± 11.6 Control: 42.3 ± 10.8	Exercise onset: 3 months after bariatric surgery. Duration: 3 days/week for 12 weeks. Protocol: 35 min of exercise (60–75%—HR reserve) and 25 min of resistance exercise (loads were not provided).	Patients received general advice regarding healthy behavior and physical activity.	SDNN, SDANN, pNN50, and RMSSD indices improved in the training group.

DNN: standard deviation of normal RR intervals; SDANN: standard deviation of the mean RR calculated over a 5 min period; RMSSD: root-mean square of differences between adjacent normal RR interval; NN50: total adjacent RR intervals with a difference in duration greater than 50 ms; pNN50: percentage of adjacent RR intervals with a difference in duration greater than 50 ms; SD1: short-term variation in the RR intervals; SD2: long-term variation in the RR intervals; RRTri: triangular index; LF: low frequency; HF: high frequency; ApEn: approximate entropy; SampEn: sample entropy.

**Table 2:** Levels of evidence analysis via (GRADE Working Group, 2004).

Certainty Assessment							No. of Patients		Effect		Certainty	Importance
No. of Studies	Study Design	Risk of Bias	Inconsistency	Indirectness	Imprecision	Other Considerations	Exercise	Control	Relative (95% CI)	Absolute (95% CI)		
Short-term variation in the RR intervals (follow-up: mean 12 weeks; assessed with: ms; scale: from 3 to 48)												
3	Randomized trials	Very serious ^a,b,c,d,e^	Very serious ^f^	Serious ^a,b,c,d,e,f^	Very serious ^g^	Publication bias strongly suspected all plausible residual confounding would suggest a spurious effect, while no effect was observed ^a,b,c,d,e,f^	20	20	-	MD 16.51 ms higher (3.58 lower to 36.59 higher)	⨁◯◯◯ Very low	CRITICAL
Root mean square of the successive differences (follow-up: mean 12 weeks; assessed with: ms; scale: from 3 to 68)												
4	Randomized trials	Very serious ^a,b,c,d,e,f^	Very serious ^f^	Serious ^a,b,c,d,e,f^	Very serious ^g^	Publication bias strongly suspected all plausible residual confounding would suggest a spurious effect, while no effect was observed ^a,b,c,d,e,f^	70	49	-	MD 8.44 ms higher (3.61 lower to 20.5 higher)	⨁◯◯◯ Very low	CRITICAL
Percentage of adjacent RR intervals with a difference in duration greater than 50 ms (follow-up: mean 12 weeks; assessed with: %; scale: from 9 to 37)												
2	Randomized trials	Very serious ^a,b,c,d,e,f^	Very serious ^f^	Serious ^b,c,d,e,f^	Very serious ^g^	Publication bias strongly suspected all plausible residual confounding would suggest a spurious effect, while no effect was observed ^b,c,d,e,h^	51	29	-	MD 13.09 ms higher (9.17 lower to 35.35 higher)	⨁◯◯◯ Very low	IMPORTANT
Standard deviation of all normal RR intervals recorded in a time interval (follow-up: mean 12 weeks; assessed with: ms; scale: from 27 to 129)												
2	Randomized trials	Very serious ^a,b,c,d,e,i^	Very serious ^i^	Serious ^a,b,c,d,e,i^	Very serious ^g^	Publication bias strongly suspected all plausible residual confounding would suggest a spurious effect, while no effect was observed ^a,b,c,d,e,g,i^	51	29	-	MD 19.74 ms higher (4.98 lower to 44.45 higher)	⨁◯◯◯ Very low	CRITICAL

CI: confidence interval; MD: mean difference. Explanations. ^a^ Allocation was not mentioned in some studies. ^b^ Exercise was not blinded. ^c^ High risk of bias in reporting bias. ^d^ High risk in other bias analysis. ^e^ Different sample size between groups. ^f^ High heterogeneity (I^2^ = 97%). ^g^ Large 95% CI. ^h^ High heterogeneity (I^2^ = 93%). ^i^ High heterogeneity (I^2^ = 85%).

## Data Availability

Data is contained within the article and Supplementary Materials.

## Supplementary Materials

The following supporting information can be downloaded at: https://www.mdpi.com/article/10.3390/jcdd11080248/s1, Risk of bias.
